# Reward and Novelty Enhance Imagination of Future Events in a Motivational-Episodic Network

**DOI:** 10.1371/journal.pone.0143477

**Published:** 2015-11-23

**Authors:** Lisa Bulganin, Bianca C. Wittmann

**Affiliations:** Department of Psychology, Justus Liebig University, Giessen, Germany; Brain and Spine Institute (ICM), FRANCE

## Abstract

Thinking about personal future events is a fundamental cognitive process that helps us make choices in daily life. We investigated how the imagination of episodic future events is influenced by implicit motivational factors known to guide decision making. In a two-day functional magnetic resonance imaging (fMRI) study, we controlled learned reward association and stimulus novelty by pre-familiarizing participants with two sets of words in a reward learning task. Words were repeatedly presented and consistently followed by monetary reward or no monetary outcome. One day later, participants imagined personal future events based on previously rewarded, unrewarded and novel words. Reward association enhanced the perceived vividness of the imagined scenes. Reward and novelty-based construction of future events were associated with higher activation of the motivational system (striatum and substantia nigra/ ventral tegmental area) and hippocampus, and functional connectivity between these areas increased during imagination of events based on reward-associated and novel words. These data indicate that implicit past motivational experience contributes to our expectation of what the future holds in store.

## Introduction

When we envision our personal future, we often imagine events as coherent scenarios unfolding in a specific place at a specific time. Imagining the future has been shown to depend on our capacity to remember past episodes, and an overlapping network of brain regions support the construction of future episodes and the recollection of the past (for a review, see [1]). The core network for both remembering and imagining consists of hippocampus and adjacent medial temporal lobe (MTL), medial prefrontal cortex (mPFC), posterior cingulate and retrosplenial (RSP) cortex, and lateral parietal and temporal regions [1].

While the importance of episodic memory to the imagination of future events is well supported, the role of implicit memory has not been addressed yet. Our daily experience is often shaped by implicitly acquired motivational values that guide decision making [2]. A central component of learned motivational value is the expectation of reward elicited by stimuli associated with reward in the past. These reward-predicting stimuli activate a network of regions receiving dopaminergic afferents from the substantia nigra / ventral tegmental area (SN/VTA), including dorsal and ventral striatum, and ventromedial PFC (vmPFC) [3,4]. Reward anticipation has been shown to exert incidental effects on a number of perceptual and cognitive processes ranging from visual attention (for a review, see [5]) to motor imagery [6], working memory (for a review, see [7]), and episodic memory (for reviews, see [8,9]). In the present study, we aimed to examine how the implicit expectation of reward elicited by conditioned reward-predicting stimuli may affect the episodic imagination of future events.

Such an influence could be mediated by interactions between the reward system and regions supporting episodic future imagination. Afferent projections to the ventral striatum include input from the vmPFC and the hippocampus, and the dopaminergic midbrain projects back to these regions (for reviews, see [10,11]). Hippocampus, striatum and SN/VTA have been shown to interact during encoding of reward and punishment-associated stimuli, promoting episodic memory (for reviews, see [8,9]). Interactions between these regions could provide a mechanism for past reward experience to modulate imagination of future events.

In addition to classical reinforcers, stimulus novelty contributes to motivated behaviour by enhancing exploration [12,13], and novel events have been shown to engage the motivational regions outlined above [14]. Novel stimuli exert a modulating influence on perceptual and cognitive processes that parallels reward effects, including effects on visual attention [15,16,17,18] and on episodic memory (for reviews, see [9,14,19]). It has been proposed that exposure to novelty could enhance future-oriented thinking and imagination, thereby increasing tonic dopaminergic firing, energizing exploratory behaviour and enhancing plasticity [14]. We therefore investigated whether stimulus novelty influences the episodic imagination of future events.

The current two-day fMRI study addressed the incidental effects of motivational memory and item novelty on the imagination of future events. On day 1, participants were pre-familiarized with two sets of words. One set was associated with reward in an instrumental task and the other set with neutral outcomes. Approximately 24 h later, we collected fMRI data during episodic future imagination based on previously rewarded, unrewarded and novel words. Participants provided ratings of task difficulty and of the vividness, coherence and valence of each imagined scene. In a post-scan interview, additional ratings were collected indicating the estimated probability of future occurrence of the imagined event. We expected future imagination based on previously rewarded words to be experienced as more vivid and positive and to involve co-activation and enhanced connectivity of hippocampus, striatum and SN/VTA compared to imagination based on neutral words. We also expected that novelty would elicit motivational effects, leading to increased vividness and higher co-activation and connectivity of hippocampus, striatum and SN/VTA during imagination based on novel compared to familiar words. Based on results reported for explicit emotional content [20], we also expected reward-based future events to be estimated as more likely to occur in the future.

## Experimental Procedures

### Participants

Thirty healthy, right-handed adults with no prior history of neurological or psychiatric disorder participated in the study. All participants gave written informed consent. The study was approved by the ethics committee of the Department of Psychology and Sports Science at the Justus Liebig University, Giessen. Data from three participants were excluded from analysis due to excessive head motion, defined as volume-to-volume motion exceeding 1 mm of translation or 1° of rotation, or motion from the first to the last volume exceeding 6 mm or 2°. Data from three other participants were excluded because of non-compliance with the instructions, and three additional participants could not be analyzed because technical problems precluded the regular completion of the experiment. The final sample comprised 21 subjects (mean age ± SD: 24.3 ± 3.4 years, 8 men). Participants were reimbursed for their participation on day 1 with the amount of money gained in the instrumental appetitive conditioning task, and on day 2 with €10 / hour.

### Stimuli

A total of 88 German nouns referring to objects were chosen from a psycholinguistic database [21] and assigned to four word lists of 22 words each. Lists did not differ significantly in word length (mean ± SD: 5.4 ± 1.6 letters) and Mannheim frequency 1,000,000 (mean ± SD: 41.5 ± 48.4) as given in the Celex database [22] nor in concreteness (mean ± SD: 8.9 ± .5), valence (mean ± SD: 5.6 ± .7), and arousal (mean ± SD: 2.5 ± .9) as rated on a 0–10 scale [21]. Example words (in translation): mirror, glove, door, letter. Assignment of word lists to experimental conditions was counterbalanced across subjects.

### Day 1: Reward task

**Fig 1:**
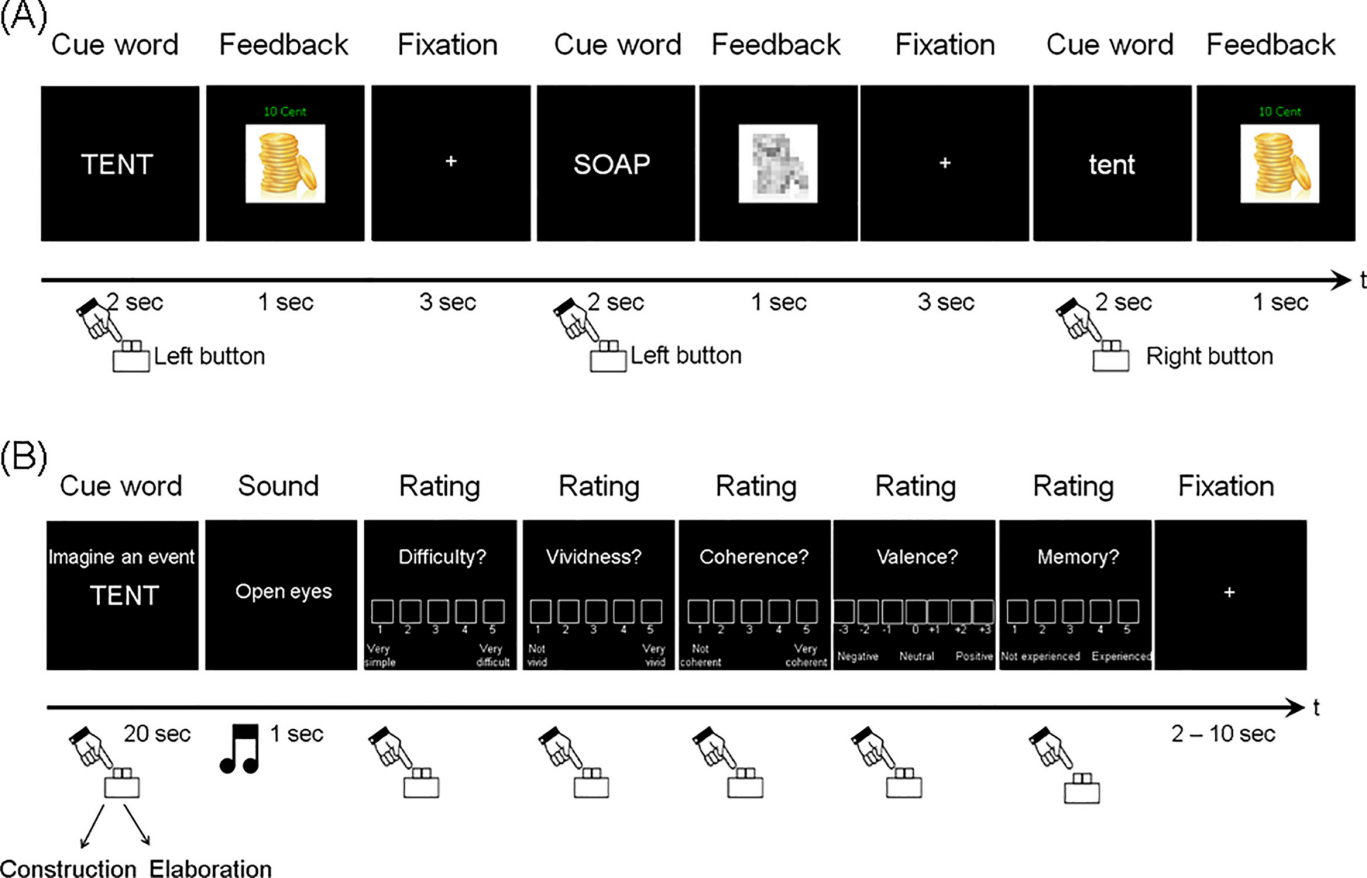
Experimental design. (A) Trial sequence for the reward task on day 1. Words were repeatedly presented to ensure familiarization and reward learning. Each word indicated whether subjects could win money for a correct response in the letter case discrimination task. In rewarded trials, subjects received win feedback (image of gold coins) for correct responses and no-win feedback (grey-scale, scrambled image) for incorrect responses. Neutral trials were always followed by no-win feedback. (B) Trial sequence for the future imagery task on day 2. A text instruction indicated the task to be performed (“imagine an event” or “find two words”). In imagery trials, participants closed their eyes after processing the instruction and cue word, constructed a future event associated with the cue word, then pressed a button and continued to imagine additional details for the event. An audio signal indicated the end of the imagery period. Subjects opened their eyes and provided self-paced ratings of the imagined event on five scales: difficulty, vividness, coherence, valence, and similarity to an existing memory.

Approximately 24 h before the fMRI session (range: 20–26 h), subjects underwent a combined familiarization and reward procedure outside the scanner (Fig 1A). Subjects were familiarized with two of the word lists by presenting each word 10 times in random order in a letter-case discrimination task (5 times in upper case and 5 times in lower case). Words from one list were associated with monetary reward, while the other list was associated with a neutral outcome. Subjects were informed that each word indicated whether reward was available for a correct response and that the mapping of each word to an outcome category (reward/neutral) remained fixed for the entire session. In each trial, subjects were shown a word centrally on the screen in white on a black background for 2 s, indicated the letter case by button press, and received feedback for 1 s, followed by an inter-trial interval (ITI) of 3 s. For correct responses to rewarded words, subjects gained €0.10 (indicated by an image of a pile of gold coins). For incorrect or missing responses to rewarded words and for all neutral words, subjects did not gain any money (indicated by a grey-scale, scrambled image of the coin pile). After every 110 trials, the task was paused until subjects were ready to continue.

### Day 2: Imagination task

Immediately before the scanning session, subjects received detailed instructions and extensive training on the episodic imagination and semantic control tasks (Fig 1B). In the scanner, subjects completed six runs of 10–12 min duration, containing a total of 22 trials for each of four conditions: Three episodic imagination conditions based on familiar rewarded (Rew), familiar neutral (Neut) and novel (Nov) words and a semantic control condition (Con) based on novel words (Methods in S1 File), which was included for comparison with previous studies [23]. Each trial started with the presentation of a two-line text cue consisting of the task instruction and a cue word (task phase, 20 s), followed by a 1 s audio tone and five rating scales (self-paced duration). Order of conditions and cue words was randomized within each run of the imagination phase. Subjects were instructed to close their eyes after reading the instruction and cue word and to keep their eyes closed until the tone announced the end of the imagination phase. Trials were separated by a jittered fixation ITI which randomly varied in duration from 2 to 10 s in 250 ms steps (mean ITI 6 s).

The episodic imagination task was signalled by the instruction “imagine an event”. Subjects were encouraged to freely associate in response to the cue word and instructed that imagined future personal events had to be plausible, probable and specific in time and place, with a maximum event duration of one day. They were instructed to imagine future events that were not an exact replication of previously experienced events and which did not involve daily routines or cultural traditions. They were also told to experience events from a field perspective rather than an observer perspective. Based on previous work, we divided the imagination phase into a construction and an elaboration phase [23]. Subjects initially generated a general framework for the future event (construction phase). When they had an event in mind, they pressed a button and then further embellished the future events with as much detail as possible until hearing the tone (elaboration phase). Subjects then rated the event on five scales: overall difficulty (1 = very easy; 5 = very difficult), vividness (1 = low in vividness and clarity; 5 = very vivid and clear), coherence (1 = low in coherence; 5 = very coherent image), valence (-3 = very negative; 0 = neutral; 3 = very positive) and use of memory (1 = not experienced in the past; 5 = exactly as experienced in the past). Each rating scale was presented on the screen and the current cursor position was highlighted. Participants used three response buttons to move the cursor and confirm the chosen rating (left button = move cursor left; middle button = move cursor right; right button = confirm rating).

In a post-scan interview conducted immediately following the fMRI session, subjects were prompted with six randomly selected cue words from each future imagination condition (27%). For each cue word, they were asked to describe the previously imagined event in detail. Subjects additionally rated each described event based on the success of adopting a field perspective (1 = almost no field perspective; 5 = very much from field perspective) and probability of event occurrence (0 = 0% probability; 10 = 100% probability). On average, they failed to recall 0.3 future events per condition (SE = 0.1), with no difference across experimental conditions (F_2,40_ = 0.69, p = .51; mean ± SE: Rew, 0.3 ± 0.1, Neut 0.2 ± 0.1, Nov 0.3 ± 0.1). The descriptions were digitally recorded, transcribed and assessed by two observers that were blind to the experimental conditions. For each event description, observers counted the number of details (such as actions, thoughts and emotions, location and external details), judged episodic specificity based on a three point scale (3 = specific in time and place; 2 = specific in either time or place; 1 = general in time and place; [23,24], and assessed overall event quality (0 = does not elicit a detailed overall picture; 10 = elicits an overall picture rich in details). Inter-rater reliability was high for number of details, specificity and quality (Cronbach’s α = .96, .86, .83, respectively).

At the end of day 2, we assessed subjects’ awareness of the reward status of words familiarized the day before. For each word, subjects indicated by button press whether the word was rewarded or neutral on day 1. Word presentation was self-paced and trials were separated by a fixation ITI (1750 ms).

### fMRI acquisition and pre-processing

Magnetic resonance images were acquired on a 1.5 T whole body scanner (Symphony, Siemens Medical Systems, Erlangen, Germany) with a standard head coil for RF transmission and signal reception. A field map was acquired with a double echo gradient echo field map sequence (TE, 10.0 and 14.76 ms; TR, 1170 ms; voxel size, 3 x 3 x 3 mm^3^, matrix size, 64 x 64) in 64 slices covering the whole head to improve distortion correction of the functional images. Functional images were acquired using blood oxygen level dependent (BOLD) signal sensitive T2*-weighted echo-planar imaging (EPI). Each volume contained 31 slices of 4 mm thickness and 3 mm in-plane resolution (TR, 2950 ms, TE, 55 ms; FOV, 192 x 192 mm; matrix size, 64 x 64) acquired in a descending sequence at a 30° angle to the anterior commissure-posterior commissure line. In each of six runs, approx. 170–240 functional whole brain volumes were collected. A T1-weighted whole-brain image (voxel size, 1.4 x 1 x 1 mm^3^) was acquired for each subject. Scanner noise was reduced with ear plugs, and subjects’ head movements were minimized with foam pads.

Raw functional data quality was checked based on volume-to-volume variance; there were no bad volumes in the final sample. Preprocessing and data analysis were performed using Statistical Parametric Mapping software implemented in Matlab (SPM8; Wellcome Trust Center for Neuroimaging, London, UK). Using the FieldMap toolbox [25,26], field maps were estimated from the phase difference between the images acquired at the short and long TE. The EPI images were corrected for distortions and for the interaction of motion and distortion based on the field map [25] using the Unwarp toolbox [26,27]. EPI images were then slice time corrected, spatially normalized to the Montreal Neurological Institute (MNI) template by segmenting and warping the acquired anatomical image to the SPM template and applying these parameters to the functional images, and smoothed using an 8 mm Gaussian kernel. A high-pass filter with a cut-off of 128 s was applied to the data.

### fMRI analysis

For analysis, only future imagination trials with vividness and coherence ratings >1 were included to ensure that events were episodic in nature. Trials that were rated as similar to existing memories (memory ratings >3) were excluded. An average of 60.6 (SD = 5.9) future imagination trials were retained for analysis, and the number of discarded trials did not differ between conditions (F_2,40_ = 0.17, p = .84; mean ± SD: Rew 20.1 ± 2.3, Neut 20.1 ± 1.9, Nov 20.3 ± 2.4). We specified the following regressors of interest for each of the four conditions (Rew, Neut, Nov, Con): onsets of the construction phase, onsets of the elaboration phase indicated by subjects’ button press (M ± SD, 4951 ± 1621 ms and 6146 ± 1796 ms for future and control task, respectively) and 2 parametric modulators for each regressor of interest (ratings of difficulty and vividness / detail). The duration of regressors of interest was set to the individual duration of the construction and elaboration phase of each trial: Construction phase duration corresponded to the time from cue onset to button press, and elaboration phase duration corresponded to the remaining time (20 s minus construction phase). For trials with no button press, the construction phase regressor was set to 20 s. There was no significant difference between conditions in the number of these trials that were included in the analysis (F2,40 = 1.43; p = 0.25; number in rew condition: 3, neut: 0, nov: 2).Parametric regressors were zero-centred and orthogonalized. Trial-related activity was modelled by convolving event regressors with a canonical hemodynamic response function [28].

A general linear model (GLM) was specified for each subject to model the effects of interest, three regressors of no interest (audio tone, rating task, excluded trials) and six covariates capturing residual motion-related artefacts. After creating voxel-wise statistical parametric maps for each subject by applying linear contrasts to the parameter estimates, a random effects analysis was performed to assess group effects. The relevant contrasts were: Neut-Con, Rew-Neut, Nov-Neut, Nov-Rew, Rew-Nov, separately for the construction and elaboration phases. Effects of difficulty and vividness were examined by specifying two separate GLMs in which difficulty (GLM1) and vividness (GLM2) were added as parametric regressors of the construction phase. Effects were first determined by pooling across conditions. Differences between conditions were then analyzed in repeated-measures ANOVAs on the betas extracted from our a priori ROIs.

Imaging results were thresholded at p < 0.005 (uncorrected) and corrected for multiple comparisons using spherical small volume correction (p < .05, family-wise error rate [FWE] corrected) for a priori regions of interest. Accordingly, for display purposes, all SVC significant activations are displayed at the initial threshold. Volumes were centred on peak voxels identified in prior motivational and episodic future imagination studies ([29,30,31], Methods in S1 File). Activations are shown overlaid onto the averaged structural MRI scan of the study participants. Stereotaxic coordinates are given in MNI space. Behavioural averages are given as mean values ± SEM except where indicated otherwise. To follow up on significant effects in repeated-measures ANOVAs, post-hoc t-tests were carried out as two-sided paired tests.

To better localize SN/VTA activity, relevant activation maps were superimposed on a mean image of the spatially normalized MT maps of 33 subjects acquired earlier [32]. MT imaging has been shown to allow distinguishing the SN from surrounding structures as a bright area, which has been confirmed to be coextensive with the SN as delineated histologically by tyrosine hydroxylase immunohistochemistry [33]. However, we will refer to BOLD activity from the entire SN/VTA complex throughout this paper because dopamine neurons are dispersed throughout the SN/VTA complex and form a functional continuum in primates [34]. This is underlined by recordings showing that dopamine neurons in the SN and VTA respond to reward [35,36].

Finally, we investigated task-modulated functional connectivity using the generalized psychophysical interaction (PPI) toolbox (gPPI) [37]. Based on anatomical connectivity between reward regions and regions in the episodic imagination network [38,39], connectivity patterns of the hippocampus, striatum and SN/VTA were investigated. For each subject, physiological time series were extracted from functionally defined ROIs in these regions (Methods in S1 File). A vmPFC seed region was not included because no reward or novelty effects were found in the vmPFC. The PPI terms were created by multiplying the deconvolved BOLD signal with the psychological regressors separately for each task condition and reconvolving with the hemodynamic response function. Second-level one-sample t-tests were carried out to reveal regions in which connectivity to the seed regions was increased during Rew vs. Neut and Nov vs. Neut trials. To investigate the networks underlying vividness differences between conditions, inter-individual difference scores were calculated from the vividness ratings by subtracting individual subjects’ mean Neut ratings from their mean Rew ratings. The resulting subject-level difference scores (range: -0.27 to 0.32) were included as a covariate in an additional analysis.

## Results

### Behavioural results

**Fig 2:**
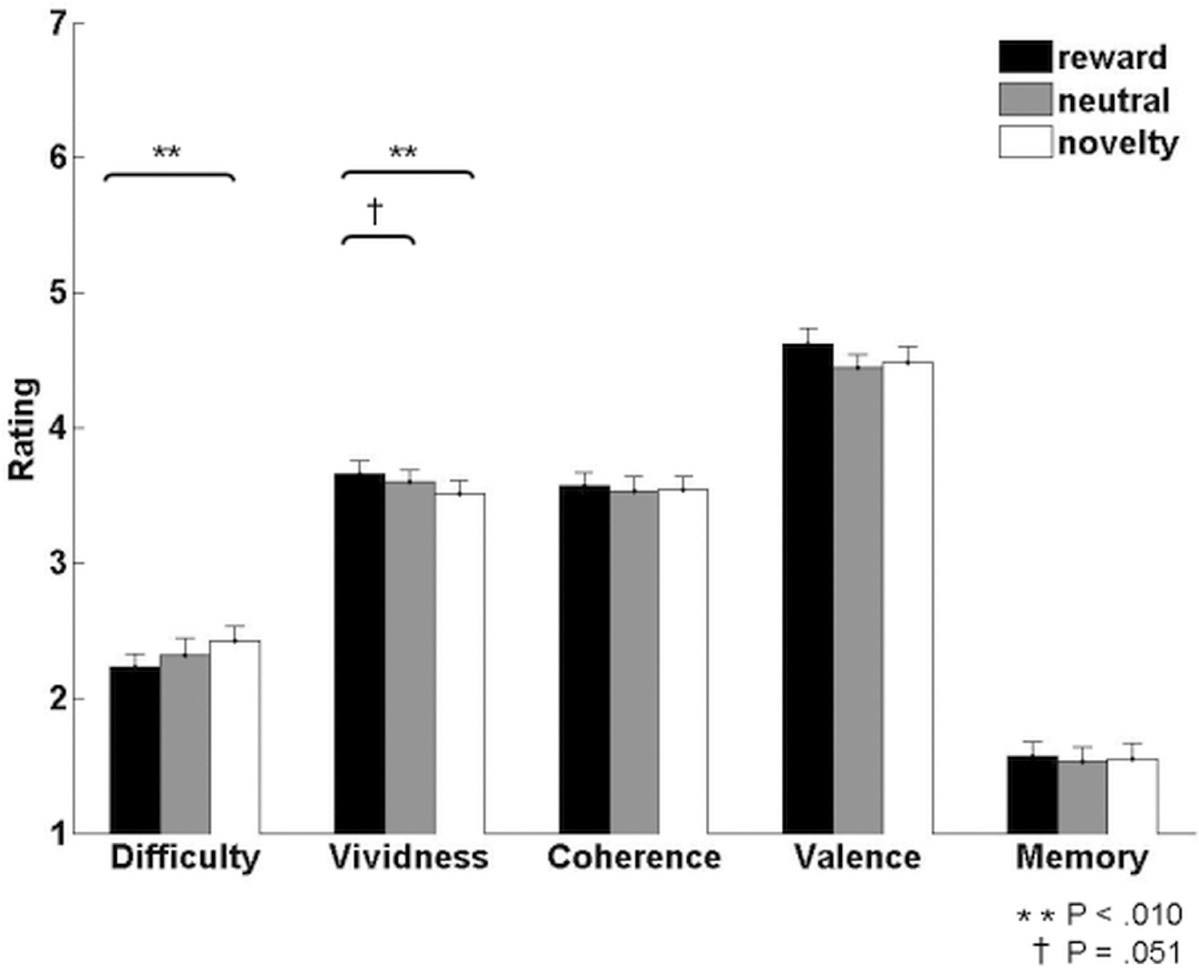
Behavioural ratings. Mean ratings of the imagined events provided by subjects. Difficulty, vividness, coherence and memory ratings were based on 5-point rating scales (1 = low, 5 = high). The valence rating was based on a scale ranging from -3 to +3, which was converted for display purposes to a scale ranging from 1 = very negative to 7 = very positive. Error bars indicate SEM.

We first tested the prediction that imagined events would be experienced as more vivid and positive in the motivational conditions compared to the neutral condition by analyzing subjects’ trial-by-trial ratings (Fig 2). Repeated-measures ANOVAs across the three imagination conditions revealed significant main effects of condition for vividness (F_2,40_ = 5.60, p = .01) and difficulty (F_2,40_ = 4.74, p = .01), while there were no significant condition effects for ratings of coherence (F_2,40_ = 0.62, p = .55), valence (F_2,40_ = 1.25, p = .30) and memory (F_2,40_ = 0.33, p = .72). Post-hoc t-tests on vividness ratings showed higher vividness for the reward compared to the novelty condition (t_20_ = 3.17, p = .01, d = 0.32) and a trend towards higher vividness for the reward compared to the neutral condition (t_20_ = 2.07, p = .051, d = 0.16), with no difference between the novelty and neutral conditions (t_20_ = -1.55, p = .14). This was not influenced by awareness of the reward status (Results in S1 File). Post-hoc paired t-tests on difficulty ratings showed significantly higher difficulty for the novelty compared to the reward condition (t_20_ = 3.01, p = .01, d = 0.40) and no significant difference between the novelty and neutral (t_20_ = 1.63, p = .12) or reward and neutral conditions (t_20_ = -1.48, p = .15). We also assessed the independence of the rating scales using multiple linear regression on the difficulty and vividness scales with the ratings on the remaining scales as independent variables. Difficulty was found to be the only significant predictor of vividness (T = -3.12, β = -.55, p = .007) and vice versa, indicating that easier trials were experienced as more vivid. Table A in S1 File shows the pairwise correlations between all scales.

We then investigated the hypothesis that imagined events based on rewarded words would be estimated as more likely to occur in the future based on ratings provided in the post-scan interview. There was a trend towards an effect of condition on ratings of future occurrence probability (F_2,40_ = 2.49, p = .096), supported by post-hoc t-tests indicating higher probability ratings for the rewarded compared to the neutral condition (t_20_ = 2.62, p = .02, d = 0.52; mean ± SE: reward, 60.5 ± 2.7%, neutral, 53.4 ± 3.4%), and no difference between the reward and novelty (mean ± SE: novelty 58.7 ± 3.9%; t_20_ = 0.53, p = .60) or novelty and neutral (t_20_ = 1.44, p = .17) conditions.

### fMRI results

To confirm the activation of the episodic construction network in our imagination task [1], we compared the neutral imagination condition to the semantic control task. Hippocampus, parahippocampal gyrus and vmPFC were activated during both phases of the imagination task (Table B in S1 File, Table E in S1 File).

#### Reward and novelty effects on future imagination

**Fig 3:**
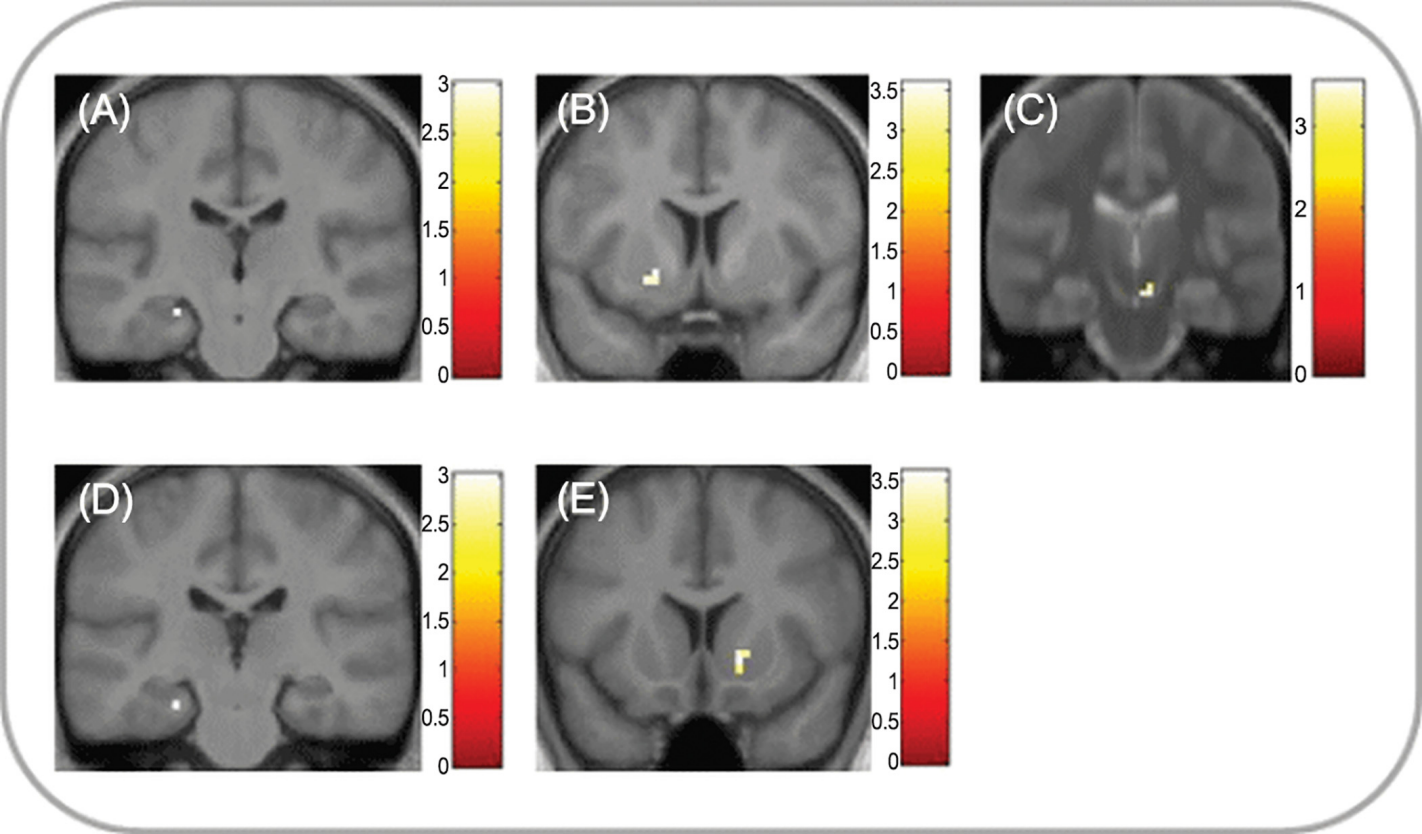
Effects of reward and novelty on the imagination of future events. (A-D) Neural activity for imagination based on rewarded words compared to familiar neutral words during the construction phase of the task. In reward-based trials, activations were significantly higher (p < .05, SVC) in (A) left hippocampus (MNI peak coordinates -24, -22, -17), (B) right mPFC (MNI 6, 53, 25), (C) left ventral striatum (MNI -18, 5, -5) and (D) right SN/VTA (MNI 6, -22, -17). (E-G) Neural activity for imagination based on novel words compared to familiar neutral words during the construction phase of the task. In novel trials, activations were significantly higher in (E) left hippocampus (MNI -24, -25, -20), (F) right mPFC (MNI 9, 50, 31), and (G) right ventral striatum (MNI 15, 11, -5). Clusters are shown in ROI masks in neurological orientation at a display threshold of p < 0.005, uncorrected. To better localize SN/VTA activations, panel D displays an overlay onto an MT image (see Experimental Procedures). Color bars indicate t values.

We then tested our hypotheses that episodic imagination based on rewarded and novel words would elicit higher co-activation of motivational regions with the episodic construction network by contrasting each motivational condition with the neutral imagination condition. Results support our hypothesis for both motivational conditions. During the construction of future events based on rewarded words, activation was significantly higher in SN/VTA, ventral striatum and hippocampus (Fig 3A–3C, Table C in S1 File). There was no significant activation in this contrast in the elaboration phase. In the novelty condition, activation was significantly higher in ventral striatum and hippocampus during the construction phase (Fig 3D and 3E, Table C in S1 File), and the ventral striatum remained significantly activated during the elaboration phase (Table C in S1 File). A combined contrast of both motivational conditions against the neutral condition confirmed activation of ventral striatum and hippocampus during the construction phase, and ventral striatum during the elaboration phase (Table C in S1 File). To further confirm the absence of SN/VTA activation in the novelty condition, we contrasted the two motivational conditions in the construction phase and found significantly higher activation in the reward condition (Table C in S1 File).

Because of the significant effect of condition on the difficulty and vividness ratings obtained in the scanner, we investigated the effect of these ratings on ROI activation during the construction phase using parametric modulation analyses. Pooling across all conditions, activation of the right striatum significantly decreased with increasing difficulty (MNI peak coordinates: 9 17–11, p(SVC) = .02), while no significant effects of vividness were found. Next, differences between conditions were analyzed in repeated-measures ANOVAs on betas extracted from the *a priori* ROIs. For vividness, activation of the left hippocampus significantly differed between conditions (F_2,40_ = 4.9, p = .01). Post-hoc t-tests revealed a stronger parametric effect of vividness in the reward condition compared to the novelty condition (t_20_ = 3.12, p = .005) and, on a trend level, compared to the neutral condition (t_20_ = 1.77, p = .09). There was a trend towards a differential effect of vividness in the striatum (F_2,40_ = 2.65, p = .08), with a higher effect for reward vs. novelty (t_20_ = 1.99, p = .06). No differences between conditions were found for difficulty.

#### Functional connectivity

**Fig 4:**
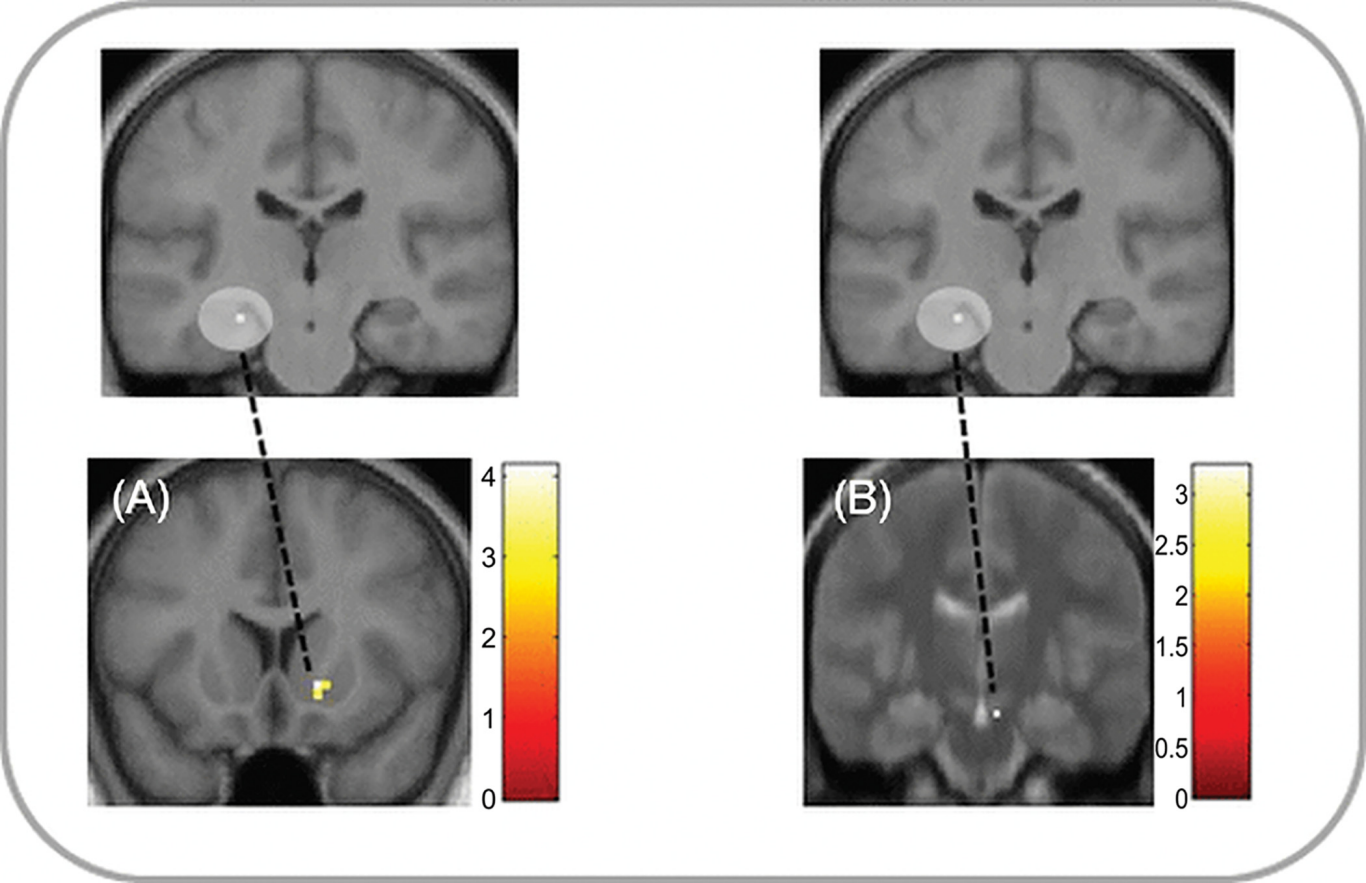
Effects of reward and novelty on functional connectivity. Significant increases in functional connectivity (p < .05, SVC) with the hippocampus during the elaboration phase for imagination based on (A) rewarded and (B) novel words compared to familiar neutral words. (A) Increased connectivity in reward compared to neutral trials between the left hippocampus seed and right ventral striatum (MNI peak coordinates 15, 14, -5). (B) Increased connectivity in novel compared to familiar neutral trials between the left hippocampus seed and (B) right SN/VTA (MNI peak coordinates 6, -16, -20). Clusters are shown in ROI masks in neurological orientation at a display threshold of p < 0.005, uncorrected. To better localize SN/VTA activations, panel B displays an overlay onto an MT image (see Experimental Procedures). Color bars indicate t values.

We next tested our prediction that functional connectivity between motivational regions and the episodic construction network should vary as a function of motivational condition by carrying out PPI analyses with seed regions in hippocampus, ventral striatum and SN/VTA. While there were no differences in connectivity during the construction phase, significant increases in hippocampal connectivity with motivational regions were found during the elaboration phase. For imagination based on rewarded words, significant functional connectivity increases were found between the hippocampus seed and ventral striatum (Fig 4A, Table D in S1 File). In the novelty condition, connectivity was significantly higher between the hippocampus seed and SN/VTA (Fig 4B, Table D in S1 File). No significant changes in connectivity were found for the striatal and SN/VTA seed in this analysis.

**Fig 5:**
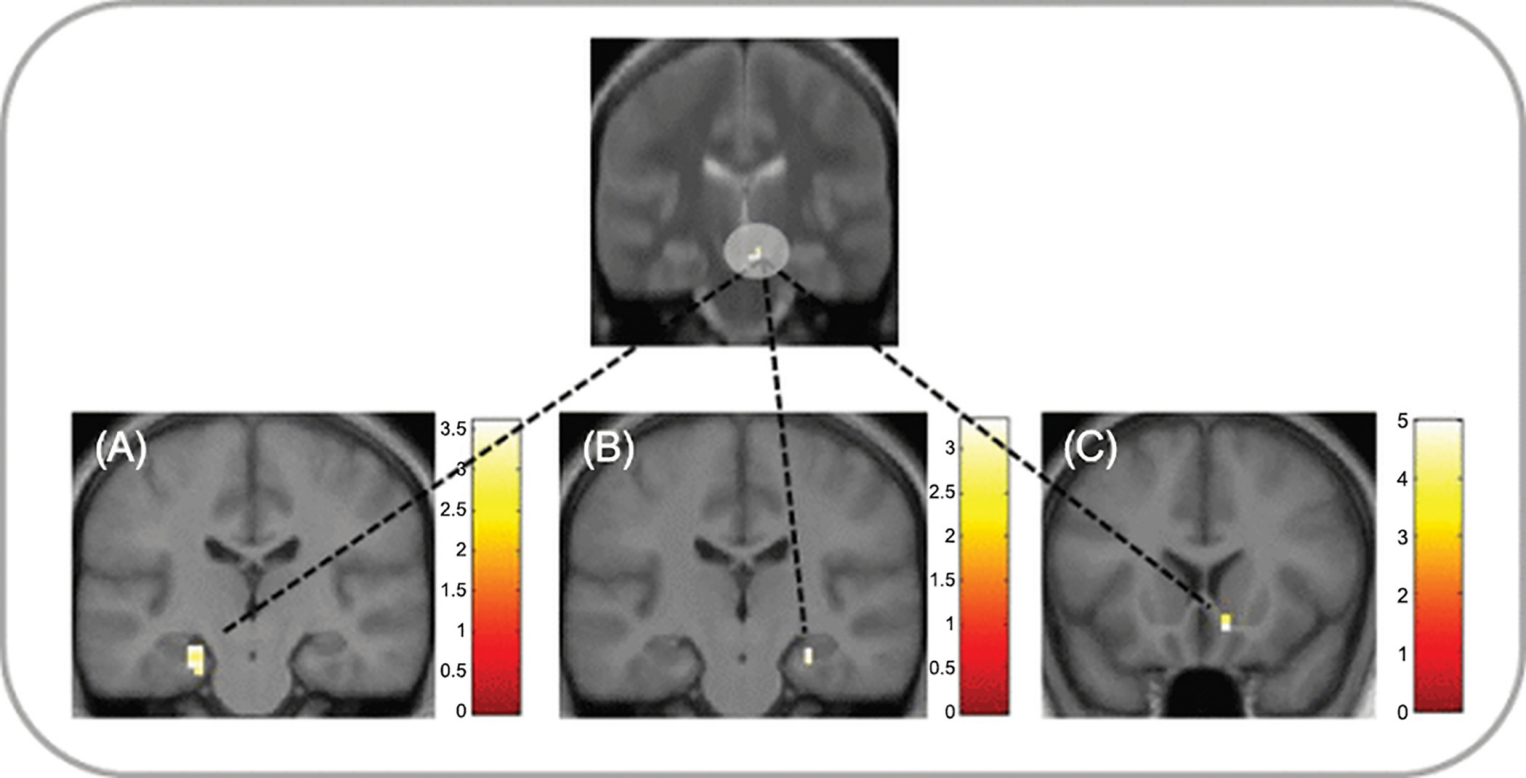
Changes in reward-associated connectivity with individual differences in vividness. In the elaboration phase, gPPI analysis revealed significant increases in functional connectivity (p < .05, SVC) in reward compared to neutral trials with increasing individual Rew-Neut difference in vividness scores between the right SN/VTA seed and (A) left hippocampus (MNI peak coordinates -24, -22, -17), (B) right hippocampus (MNI 27, -19, -17), and C) right ventral striatum (MNI 9, 17, -11). Clusters are shown in ROI masks in neurological orientation at a display threshold of p < 0.005, uncorrected. To better localize SN/VTA activations, the top panel displays an overlay onto an MT image (see Experimental Procedures). Color bars indicate t values.

To investigate the neural basis of the observed difference in vividness ratings between conditions, we next included individual difference scores (Rew–Neut vividness scores) as a second-level covariate. Difference scores correlated with enhancement of SN/VTA-hippocampal and SN/VTA-striatal functional connectivity during reward-based imagination (Fig 5, Table D in S1 File). No significant changes in connectivity related to individual difference scores were found for the striatal and hippocampus seed in this analysis.

## Discussion

Our data show that prior reward association and novelty enhance the imagination of future personal events. Imagination based on previously rewarded words was rated as more vivid and was associated with co-activation of hippocampus, striatum and SN/VTA during event construction and with enhanced functional connectivity between these regions during event elaboration. Co-activation and connectivity of these areas also increased during imagination based on novel words, suggesting that novelty enhanced future thinking as a result of its motivational properties. These results are consistent with the hypothesis that the imagination of personal future events is enhanced for events constructed around novel stimuli and stimuli associated with past reward experience.

Imagining the future depends on flexibly accessing past experiences, including explicit representation of affective content [40,41]. Based on known effects of reward on cognition [6,7,8], we expected that implicit reward association would similarly modulate imagination of future events by increasing the vividness and positive valence of the imagined events. In line with this hypothesis, vividness of reward-based imagined scenes was higher in the reward condition at a strong trend level (Fig 2), indicating that reward association led to greater clarity and vibrancy of the imagined scene. An alternative interpretation of these effects could be that the increased perception of vividness was based on increased memory encoding of the imagined scenes, since previous studies found that incidental reward enhances memory encoding in the absence of an imagination task [29,42] and that highly detailed imagined events were more likely to be encoded [43,44]. This interpretation would also be consistent with the increased activation of the hippocampus in the reward condition. However, although the current study was not specifically designed to test memory, results from the post-scan interview suggest that changes in vividness and hippocampal activations did not result from reward effects on memory, since recall of imagined events did not differ between the three conditions. There remains a possibility that prior reward association could have selectively affected memory consolidation, which was not tested in this study. The possibility of encoding-related effects is also not supported by the parametric modulation analysis. While increased encoding would be expected in both the reward and novelty condition [8], vividness selectively modulated hippocampal activity in the reward condition compared to the novelty condition, suggesting specific effects of reward on future imagination.

Our results support the hypothesis that reward experience could affect future-directed behaviour through a change in expectation of future occurrence, as indicated by the higher occurrence probability estimates. Previous studies found higher probability ratings for emotionally positive or negative future events that were repeatedly imagined [20], and past events with positive valence were estimated as more probable to re-occur in the future [45]. Our results now extend these findings from explicit representation of emotional content to implicit effects of prior reward association, as valence ratings did not differ across conditions in the current study. These valence results do not support our initial hypothesis that reward-based future events would be perceived as more positive. However, the current results are consistent with the idea that reward learning and motivational processes can be dissociated from hedonic experience [46]. In line with Szpunar and Schacter’s (2013)[20] finding that increases in probability ratings across repeated imagination were correlated with changes in ease, detail and arousal, the higher probability estimates for reward-based imagination could reflect the increased vividness and ease in the reward condition. This concurrent increase in vividness and probability estimates could also be interpreted as reflecting a higher optimism bias in the reward condition. More optimistic subjects have been shown to report a greater sense of pre-experiencing positive events [47], and optimism is higher in subjects reporting higher vividness of imagining positive future events [48]. In a study on belief updating and optimism bias, greater updating in a desirable direction was observed after treatment with levodopa [49], suggesting that optimism can be influenced by mesolimbic reward learning mechanisms. This is also consistent with the finding that levodopa administration during imagination of positive future events increased participants’ subsequent estimates of the hedonic value of these events [50], which is known to involve the striatum [51]. The current results suggest an involvement of the mesolimbic system in mediating the vividness of imagination based on previous reward experience. A caveat regarding the probability estimates is that only a subset of events could be included in the post-scan test because of time constraints.

The current results also support the hypothesis that prior reward association enhances activation in the hippocampus, which plays a central role in imagining the future [1], as well as in SN/VTA and striatum, two main areas associated with reward processing [3,4]. Consistent with predominantly phasic effects of reward [9,52], Imagination of future events based on reward-associated words compared to familiar neutral words affected motivational and episodic activations during event construction, but not during event elaboration. Most of the previous fMRI studies investigating the modulation of future imagination by contextual or emotional factors did not separate event construction and elaboration [41,53,54]. Other studies found an effect of event specificity on hippocampal activation during event construction but not elaboration [55], and higher hippocampal activation at event construction for imagined events that were successfully encoded into memory [43]. Taken together, these prior studies and the current results suggest that modulatory effects on future imagination could be more pronounced during the initial generation of a scene. Scene construction is a central component of imagining future events that relies on hippocampal contributions to recombination and binding of details into a coherent spatiotemporal context [1,31,56]. This initial process of retrieving and recombining details from memory may be more open to modulatory factors than the subsequent inclusion of additional details, which may more logically follow from the initial event setup.

Our findings are consistent with the expectation that novel stimuli would enhance future imagination through increased activity in the motivation network. Novelty signals the availability of action opportunities potentially relevant for survival, enhances exploratory behaviour [12,13] and has been suggested to enhance future-oriented thinking and imagination [14]. In accordance with this, imagination of future events based on novel words elicited higher activity in striatum and hippocampus (Fig 3), suggesting that the motivational properties of novelty enhanced future-directed thinking. In contrast to reward, however, novelty-based event construction did not activate the SN/VTA. This is an unexpected finding given that the SN/VTA is a central component of a hippocampal-VTA loop mediating encoding of novel events into long-term memory based on dopaminergic input into the hippocampus [9,14,19]. However, while some fMRI studies found activation of the SN/VTA by novel stimuli and novelty anticipation [32,57,58], other studies did not report midbrain activation by novelty [30,59,60,61]. These differences could be due to task effects. In the current study, the overall task-related orientation towards construction of novel future events may have interacted with stimulus novelty, reducing differences in midbrain activation between the novel and familiar neutral conditions [14]. A previous study investigated the effect of pre-existing familiarity with elements of the imagined scene, reporting higher vmPFC activation with increasing familiarity of elements from everyday life [40]. There was no effect of decreasing familiarity of the elements, presumably because all elements were familiar to the subjects. Future studies could address the effects of different tasks and stimulus categories on activity in SN/VTA, striatum and hippocampus, the main components of the hippocampal-VTA loop [19]. A potential confound for the novelty contrast in the current study is the familiarization procedure for the neutral words, which may have come to be associated with learned irrelevance after being presented together with reward-predicting words on day 1. However, this arguably reflects the acquisition of neutral value in the real world, where organisms constantly face a mix of stimuli signalling motivational and non-motivational events.

While reward effects were specific to the construction phase of the task, novelty effects were found in both task phases. In addition to the effects during event construction discussed above, the ventral striatum was activated during the elaboration phase compared to the neutral condition. This is consistent with animal studies showing that novelty affects tonic firing of dopaminergic neurons [19,62] and thereby leads to sustained dopamine release in striatum [63,64] and hippocampus [65] in contrast to phasic reward responses [9,52]. We also found increased connectivity between the hippocampus and the SN/VTA during novelty-based compared to familiarity-based event elaboration (Fig 4, Table D in S1 File), supporting the idea of extended novelty effects in the hippocampal-VTA loop [9,19]. Enhanced functional connectivity could be based on direct anatomical connections that exist between hippocampus, striatum and SN/VTA [10,11]. Hippocampal connectivity during event elaboration was also higher for reward-based compared to neutral imagination (Fig 4, Table D in S1 File). It is possible that this reflected retrieval of motivational, action-related details that could increase the perceived vividness of the imagined event. This is supported by the correlation of individual difference scores in vividness between reward and neutral trials with enhanced SN/VTA-hippocampal and SN/VTA-striatal connectivity (Fig 5, Table D in S1 File), suggesting an underlying neural mechanism for behavioural effects of reward on future imagination.

A central role of imagining the future lies in allowing us to prepare for important events (for a review, see [1], for example by promoting future-directed decisions [66,67]. This suggests a possible relevance of the current findings for future-oriented choice. Reward and novelty constitute important elements of goal-directed behaviour and decision making [2], which could potentially bias choice by modulating imagination processes. When choosing between future options, we engage mental simulation processes to facilitate decision making [68,69]. The reward-associated increase in vividness in our study may constitute a way through which past reward experience influences future-directed choice, consistent with previous studies on the influence of future imagination on intertemporal choice that found lower discounting of future options with increasing trial-by-trial ratings of the emotional intensity of episodic imagination [66] and reported that individual differences in frequency and vividness of episodic associations across subjects were correlated with individual discount rates in intertemporal choice [67]. This is also in line with evidence that higher vividness of episodic imagination increases action tendencies related to the imagined episode [70]. Additional influence on choice could arise from the reward effect on probability estimates of the imagined events. Since probability estimates are taken into account in decision making, e.g. by calculating expected value based on reward probability and magnitude [71], a change in probability estimates can be expected to directly influence choice. Novelty has also been suggested to exert some of its behavioural effects via an enhancement of future-oriented imagination [14], an idea that is supported by the overall novelty-based enhancement of episodic future imagination processes in the current study, suggesting a potential mechanism for increased engagement with future action possibilities and thus for promoting choices of less familiar courses of action.

Our results show that the motivational factors reward and novelty enhance episodic imagination of the future. Through modulation of motivational-episodic networks, our past experience may implicitly bias our expectation of personal future events. Future studies could address subsequent effects on future-directed choices arising from these reward effects on imagination.

## Supporting Information

S1 File Supplementary text and tables. (DOC)
