# Supplementary material for: Reward and Novelty Enhance Imagination of Future Events in a Motivational-Episodic Network
Source: PLoS One. 2015 Nov 23;10(11):e0143477. doi: 10.1371/journal.pone.0143477 (PMC4657902; doi:10.1371/journal.pone.0143477)
Supplement: S1 File — (DOC) [file pone.0143477.s001.doc]

**Supplementary Text**

**Methods**

*Semantic control task*

The semantic control task was signalled by the instruction “find two words”. Subjects were instructed to find two nouns that were semantically related to the cue word, generate a sentence containing all three words, and then press a button (construction phase). They then had to think of additional meanings of the three words until hearing the tone (elaboration phase). At the end of each trial, subjects rated the semantic content on five scales: difficulty (1 = very easy; 5 = very difficult), amount of detail (1 = few details; 5 = very many details); semantic relatedness (1 = unrelated; 5 = closely related), valence of the two generated words (-3 = very negative; 0 = neutral; 3 = very positive) and meaningfulness of the generated sentence (1 = not meaningful; 5 = very meaningful). This task includes generative processes such as semantic retrieval and associative processes that are also required for future imagery. In the current study, it was included to assess whether episodic future imagery activated the same network as in previous studies .

*A priori ROI definition*

Volumes were centred on peak voxels identified in prior motivational and episodic future imagination studies . Reward-associated peak coordinates in ventral striatum (MNI coordinates: -15.15, 11.73, -8.85) and SN/VTA (6.06, -21, -15.51) were based on Wittmann et al. (2005). The novelty-associated ventral striatum peak (10, 16, -2) was defined based on Guitart-Masip et al. (2010). Coordinates for hippocampus (24, -21, -15; -21, -21, -21), parahippocampal gyrus (21, -33, -18; -18, -33, -15) and vmPFC (-3, 48, -12) were taken from Hassabis et al. (2007). SVC radii corresponded to the regional anatomical volumes: ventral striatum (9 mm ), SN/VTA (4.5 mm ), hippocampus (6 mm ), parahippocampal cortex (8.6 mm ) and vmPFC (15 mm ).

*ROI definition for PPI analyses*

For each subject, physiological time series were extracted from functionally defined ROIs in the hippocampus, SN/VTA and striatum. For the hippocampus, we were able to determine individual peak voxels from 20 subjects, with a maximum deviation of x = 6 mm, y = 15 mm and z = 9 mm from the group peak identified in the contrast of all future imagery conditions vs. the semantic control condition (left hippocampus: ‑24 ‑22 ‑17). Because the SN/VTA and striatum were not activated in this contrast, ROIs were defined based on the contrast of the reward vs. neutral condition. For the striatum, we were able to determine individual peak voxels from 20 subjects, with a maximum deviation of x = 12 mm, y = 22 mm and z = 18 mm from the group peak (left striatum: ‑18 5 ‑5). Because of the small size of the SN/VTA, time series for all subjects were extracted centred on the group peak (right SN/VTA: 6 ‑22 ‑17). The mean of each regional time series was calculated across all voxels in a 3 mm sphere centred on the respective peak voxel. The results of these analyses were tested using the ROI approach described for the main contrasts (see Experimental Procedures).

**Behavioural Results**

*Reward task*

In the reward task on day 1, performance in the letter case discrimination task did not differ between rewarded and neutral trials (mean: rewarded, 99.18 ± 0.16 %, neutral, 99.05 ± 0.17 %, t_20_ = 0.84, p = .41). In a 2 x 2 ANOVA (reward status x letter case) on reaction time, there was no main effect of reward status (F_1,20_ = 0.35, p = .56) or letter case (F_1,20_ = 0.85, p = .37) and no interaction (F_1,20_ < 0.001, p = .99; mean ± SE: reward/upper-case 585 ± 19 ms; reward/lower-case 593 ± 16 ms; neutral/upper-case 586 ± 19 ms; neutral/lower-case 595 ± 18 ms).

At the end of day 2, we tested subjects’ awareness of the reward status of familiarized words (see Experimental Procedures). A paired t-test confirmed that participants had learned the reward status and that awareness did not differ between Rew and Neut (t_20_ = -0.14, p = .89; mean percentage correct ± SE: reward, 75.1 ± 3.7 %, neutral, 75.5 ± 3.5 %).

*Imagination task*

We investigated differences in the duration of the construction phase between the imagination and control conditions. A one-way repeated-measures ANOVA revealed a significant effect (F_3,60_ = 18.4, p < .001), which was further probed in post-hoc comparisons. Pairwise t-tests revealed significantly longer duration of the construction phase in the control condition compared to the imagination conditions (rew > control t_20_ = -4.08, p = 0.001; neut > control t_20_ = -5.14, p < 0.001; nov > control t_20_ = -4.65, p < 0.001). There were no significant differences between any of the imagination conditions (all p > 0.8), confirming that differences in task difficulty did not affect the duration of task phases in the imagination task. Subjects required an average of 4.95 s (SD = 1.62 s) to construct a future event, and 6.15 s (SD = 1.8 s) to generate a sentence in the control task. In addition, there was no correlation between the duration of the construction phase in the imagination conditions with ratings of difficulty (r = -.155, p = .50) or vividness (r = -.07, p = .76).

In an additional ANOVA, we investigated whether the difference in vividness ratings was due to subjects’ awareness of the reward status of the words. There was no interaction in ratings between true reward status and subjects’ awareness (F_1,18_ = 0.3; p = .59).

*Post-scan event descriptions*

We assessed subjects’ compliance with the instruction to imagine events from a field perspective through post-scan ratings (1-5 scale). A repeated-measures ANOVA across the three imagery conditions revealed that participants successfully viewed items from a field perspective, with no difference in field perspective ratings across conditions (F_2,40_ = 0.36, p = .70; mean ± SE: Rew 4.3 ± .1, Neut 4.2 ± .1, Nov 4.2 ± .1).

Two observers that were blind to event condition rated the event descriptions from the post-scan interview for number of details, overall quality (0-10 scale) and episodic specificity (1-3 scale). As interrater reliability was high for number of details, specificity and quality (Cronbach’s α = .96, .86, .83, respectively), the mean across both observers was entered into further analyses. Repeated-measures ANOVAs across the three conditions showed no difference in the mean number of details (F_2,40_ = 0.70, p = .50; mean ± SE: Rew 30.5 ± 1.7; Neut 28.7 ± 1.9; Nov 29.8 ± 1.8) and no difference in overall event quality ratings (F_2,40_ = 0.83, p = .44; mean ± SE: reward 6.2 ± 0.2; neutral 6.0 ± 0.2; novelty 6.1 ± 0.2). Events from all conditions were also rated as highly specific in time and place (F_2,40_ = 0.67, p = .52; mean ± SE: reward: 2.9 ± 0.03; neutral 2.9 ± 0.04; novelty 2.9 ± 0.04).

**Supplementary References**

1. Addis DR, Wong AT, Schacter DL. Remembering the past and imagining the future: common and distinct neural substrates during event construction and elaboration. Neuropsychologia. 2007;45(7): 1363-77

2. Wittmann BC, Schott BH, Guderian S, Frey JU, Heinze HJ, Duzel E. Reward-related FMRI activation of dopaminergic midbrain is associated with enhanced hippocampus-dependent long-term memory formation. Neuron. 2005;45(3): 459-67.

3. Guitart-Masip M, Bunzeck N, Stephan KE, Dolan RJ, Duzel E. Contextual novelty changes reward representations in the striatum. J Neurosci. 2010;30(5): 1721-6.

4. Hassabis D, Kumaran D, Maguire EA. Using imagination to understand the neural basis of episodic memory. J Neurosci. 2007;27(52): 14365-74.

5. Anastasi G, Cutroneo G, Tomasello F, Lucerna S, Vitetta A, Bramanti P, et al. In vivo basal ganglia volumetry through application of NURBS models to MR images. Neuroradiology. 2006;48(5): 338-45.

6. Geng DY, Li YX, Zee CS. Magnetic resonance imaging-based volumetric analysis of basal ganglia nuclei and substantia nigra in patients with Parkinson's disease. Neurosurgery. 2006;58(2): 256-62

7. Lupien SJ, Evans A, Lord C, Miles J, Pruessner M, Pike B, et al. Hippocampal volume is as variable in young as in older adults: implications for the notion of hippocampal atrophy in humans. Neuroimage. 2007;34(2): 479-85.

8. Pruessner JC, Kohler S, Crane J, Pruessner M, Lord C, Byrne A, et al. Volumetry of temporopolar, perirhinal, entorhinal and parahippocampal cortex from high-resolution MR images: considering the variability of the collateral sulcus. Cereb Cortex. 2002;12(12): 1342-53.

9. Hesslinger B, Tebartz van Elst L, Thiel T, Haegele K, Hennig J, Ebert D. Frontoorbital volume reductions in adult patients with attention deficit hyperactivity disorder. Neurosci Lett. 2002;328(3): 319-21.

**Supplementary** **Tables**

Supplementary Table A. Pairwise correlations between event ratings

|  | Difficulty | Vividness | Coherence | Valence | Memory |
| --- | --- | --- | --- | --- | --- |
| Difficulty | - |  |  |  |  |
| Vividness | -.83*** | - |  |  |  |
| Coherence | -.73*** | .81*** | - |  |  |
| Valence | -.41 | .39 | .38 | - |  |
| Memory | .29 | -.49* | -.59** | -.26 | - |

Pearson correlations for ratings of the imagined events. *p<0.05, **p<0.01, ***p<0.001

Supplementary Table B. Future imagery network

| **Region** | **Hemisphere** | **MNI coordinates  (x, y, z)** | **Z-score** | **p-value*** |
| --- | --- | --- | --- | --- |
| Construction phase |  |  |  |  |
| Ventromedial PFC | R | 3, 56, -5 | 4.54 | 0.001 |
| Ventromedial PFC | L | -3, 38, -14 | 5.36 | <0.001 |
| Hippocampus | R | 24, -22, -20 | 4.48 | <0.001 |
| Hippocampus | L | -21, -16, -20 | 3.50 | 0.006 |
| Parahippocampal gyrus | R | 24, -34, -17 | 4.08 | 0.002 |
| Parahippocampal gyrus | L | -27, -37, -11 | 5.31 | <0.001 |
| Elaboration phase |  |  |  |  |
| Ventromedial PFC | L | -9, 50, -8 | 5.33 | <0.001 |
| Hippocampus | R | 24, -25, -17 | 2.73 | 0.041 |
| Parahippocampal gyrus | R | 24, -34, -14 | 4.89 | <0.001 |
| Parahippocampal gyrus | L | -21, -37, -14 | 3.49 | 0.012 |

Peak MNI coordinates and z statistics for all a priori regions significantly activated during future imagery based on neutral words compared to the semantic control task. *FWE-corrected in a priori ROIs.

Supplementary Table C. Reward and novelty effects on future imagery

| **Region** | **Hemisphere** | **MNI coordinates  (x, y, z)** | **Z-score** | **p-value*** |
| --- | --- | --- | --- | --- |
| Rew > Neut, Construction phase |  |  |  |  |
| Hippocampus | L | -24, -22, -17 | 2.72 | 0.050 |
| Parahippocampal gyrus | L | -21, -34, -17 | 3.27 | 0.028 |
| Ventral striatum | L | -18, 5, -5 | 3.12 | 0.048 |
| Substantia nigra/ventral tegmental area | L | 6, -22, -17 | 3.08 | 0.011 |
| Nov > Neut, Construction phase |  |  |  |  |
| Hippocampus | L | -24, -25, -20 | 2.72 | 0.046 |
| Parahippocampal gyrus | R | 27, -34, -23 | 3.06 | 0.042 |
| Ventral striatum | R | 15, 11, -5 | 3.13 | 0.044 |
| Nov > Neut, Elaboration phase |  |  |  |  |
| Ventral striatum | R | 6, 20, -2 | 4.40 | 0.001 |
| (Rew + Nov) > Neut, Construction phase |  |  |  |  |
| Hippocampus | L | -24, -22, -17 | 2.87 | 0.033 |
| Ventral Striatum | R | 15, 11, -2 | 3.13 | 0.046 |
| Ventral Striatum | L | -9, 17, -11 | 3.18 | 0.039 |
| (Rew + Nov) > Neut, Elaboration phase |  |  |  |  |
| Ventral Striatum | R | 3, 17, -2 | 4.34 | 0.001 |
| Rew > Nov, Construction phase |  |  |  |  |
| SN/VTA | R | 6, -19, -17 | 2.60 | 0.037 |

Peak MNI coordinates and z statistics for all a priori regions with significant effects of reward and novelty. *FWE-corrected in a priori ROIs.

Supplementary Table D. Functional connectivity during the elaboration phase

| **Region** | **Hemisphere** | **MNI coordinates  (x, y, z)** | **Z-score** | **p-value*** |
| --- | --- | --- | --- | --- |
| Rew > Neut, Hippocampus seed |  |  |  |  |
| Ventral striatum | R | 15, 14, -5 | 3.44 | 0.028 |
| Nov > Neut, Hippocampus seed |  |  |  |  |
| Substantia nigra/ventral tegmental area | R | 6, -16, -20 | 2.89 | 0.023 |
| Retrosplenial cortex | R | 12, -52, 7 | 2.73 | 0.023 |
| Rew > Neut correlation with individual difference in Rew-Neut vividness scores, SN/VTA seed |  |  |  |  |
| Ventral striatum | R | 9, 17, -11 | 3.94 | 0.004 |
| Hippocampus | R | 27, -19, -17 | 2.92 | 0.040 |
| Hippocampus | L | -24, -22, -17 | 3.11 | 0.024 |

Peak MNI coordinates and z statistics for all a priori regions with significant functional connectivity changes to one of the seed regions during the elaboration phase. *FWE-corrected in a priori ROIs.

Supplementary Table E. Functional imaging results outside a priori ROIs

| **Region** | **Hemisphere** | **MNI coordinates  (x, y, z)** | **Z-score** | **p-value*** |
| --- | --- | --- | --- | --- |
| Rew > Neut, Construction phase phaphase |  |  |  |  |
| Postcentral gyrus | L | -33, -37, 55 | 4.77 | 0.049 |
| Nov > Neut, Elaboration phase |  |  |  |  |
| Posterior cingulate | R | 9, -34, 28 | 4.87 | 0.029 |
| Neut > Con, Construction phase |  |  |  |  |
| Precuneus | R | 3, -52, 16 | 6.17 | <0.001 |
| Ventromedial PFC | L | -3, 38, -14 | 5.36 | 0.002 |
| Lateral parietal lobule | L | -48, -73, 31 | 5.06 | 0.011 |
| Middle temporal gyrus | R | 60, -4, -20 | 4.83 | 0.035 |
| Cerebellum | L | -3, -52, -44 | 4.69 | 0.073 |
| Superior frontal gyrus | L | -18, 35, 43 | 4.67 | 0.080 |
| Neut > Con, Elaboration phase |  |  |  |  |
| Middle temporal gyrus | R | 57, -4, -20 | 5.34 | 0.002 |
| Ventromedial PFC | L | -9, 50, -8 | 5.33 | 0.003 |

Peak MNI coordinates and z statistics for regions significant in a whole-brain analysis at an exploratory threshold of p<.01. *FWE-corrected at the whole-brain level
